# Supplementary material for: A Proposal for a New Lung Ultrasound Score in Rheumatoid Arthritis: The Reliability of Lung Ultrasound for Rheumatoid Arthritis-Associated Interstitial Lung Disease Diagnosis
Source: J Clin Med. 2025 May 25;14(11):3701. doi: 10.3390/jcm14113701 (PMC12156292; doi:10.3390/jcm14113701)
Supplement: Supplementary file 1 [file jcm-14-03701-s001.zip › jcm-3600779-supplementary.pdf]

Table S1. Main characteristics of patients with suspected RA-ILD.

Variables	Patients with RA (n=14)
**Socio-demographic**	
Age, mean ± SD	68.2 ± 8.1
Sex, female, n (%)	12 (85.7)
Smoking habit, n (%)	5 (35.7)
**Disease related measures**	
Rheumatoid factor, positive, n (%)	14 (100)
ACPA, n (%)	13 (92.9)
DAS28, mean ± SD	3.1 ± 1.3
HAQ, mean ± SD	0.438 ± 0.654
**Respiratory and systemic symptoms**	
Dyspnoea, n (%)	8 (57.1)
Persistent cough, n (%)	1 (57.1)
Fatigue, n (%)	6 (42.9)
**Therapies**	
Methotrexate, n (%)	10 (71.3)
Other csDMARDs, n (%)	9 (64.3)
TNF antagonists, n (%)	3 (21.4)
Other biologics, n (%)	5 (35.7)
Glucocorticoids, n (%)	6 (42.9)

ACPA: anti-citrullinated peptide antibodies; csDMARDs: conventional synthetic disease modifying antirheumatic drugs; DAS28: Disease Activity Score of 28 joints; HAQ: Health Assessment Questionnaire; ILD: interstitial lung disease; n: number; RA: rheumatoid arthritis; SD: standard deviation; TNF: tumor necrosis factor.
